# The role of reproductive isolation in allopolyploid speciation patterns: empirical insights from the progenitors of common wheat

**DOI:** 10.1038/s41598-017-15919-z

**Published:** 2017-11-22

**Authors:** Yoshihiro Matsuoka, Shigeo Takumi

**Affiliations:** 1 Fukui Prefectural University, Matsuoka, Eiheiji, Yoshida, Fukui, 910-1195 Japan; 2 Graduate School of Agricultural Science, Kobe University, Nada-ku, Kobe, 657-8501 Japan

## Abstract

The ability to cause reproductive isolation often varies among individuals within a plant species. We addressed whether such polymorphism influenced speciation of the allopolyploid common wheat (*Triticum aestivum* L., AABBDD genome) by evaluating the expression of pre-pollination (outcrossing potential) and post-pollination (crossability) barriers in *Aegilops tauschii* Coss. (the D genome progenitor). In total, 201 *Ae. tauschii* accessions representing the entire natural habitat range of the species were used for anther length measurement and artificial crosses with a *Triticum turgidum* L. (the AB genome progenitor) tester. Intraspecific comparisons showed that both barriers were more strongly expressed in the TauL1 lineage than in the TauL2 lineage. The ability of *Ae. tauschii* to cause reproductive isolation in the hybridisation with *T. turgidum* might have markedly influenced common wheat’s speciation by inducing lineage-associated patterns of gene flow. The TauL2 accessions with high potential for natural hybridisation with *T. turgidum* clustered in the southern coastal Caspian region. This provided phenotypic support for the derivation of the D genome of common wheat from southern Caspian populations. The present study underscored the importance of approaches that incorporate the genealogical and geographic structure of the parental species’ reproductive isolation in understanding the mechanism of plant allopolyploid speciation.

## Introduction

Allopolyploidy, defined as the merging of different chromosome sets through hybridisation, is an important driver of plant speciation and diversification^1–3^. How allopolyploid species form is a long-standing question. In contrast to the recent advances in understanding the nuclear and cellular level changes in emerging allopolyploids, much remains uncertain about the genetic and ecological basis of allopolyploid speciation^4–7^. Natural allopolyploids may arise through successful species crossing followed by hybrid genome duplication via the union of unreduced gametes, when related but diverged species come into contact due to migration, habitat disturbance, or distribution range shifts. Among other factors, the two mechanisms that act in opposite directions, namely reproductive isolation between the parental species and unreduced gamete production in their hybrids, strongly influence the early stages of allopolyploidy^8–10^.

Recent studies increased our knowledge on the genetic mechanisms that underlie reproductive isolation in plants. Types, functions, and causal mutations/variations are now known for several pre-zygotic and post-zygotic barrier genes that reduce the amount of gene flow between species or populations in model and non-model systems^11–14^. The mechanism underlying unreduced gamete production in hybrids is largely unknown, but a few studies addressed its genetic basis^15,16^. Importantly, the parental plant species are usually polymorphic in their ability for reproductive isolation and for hybrids’ unreduced gamete production. Thus, some parental individuals may be involved in natural allopolyploid formation more often than others may because they frequently hybridise and because their hybrids are likely to produce unreduced gametes. Such polymorphism may have profound impact on the temporal and spatial patterns of allopolyploid speciation particularly when it is genealogically and/or geographically structured. Nevertheless, the influence of such polymorphism on allopolyploid speciation remains understudied.

The present study aimed to provide novel insights into the role of reproductive isolation during the allopolyploid speciation of common wheat (*Triticum aestivum* L., AABBDD genome). Our goal was to elucidate how the diploid progenitor’s ability to cause early reproductive isolation in hybridisation with the tetraploid progenitor influenced the allopolyploid speciation of common wheat. For this goal, we analysed the patterns of the expression of pre-pollination (outcrossing potential) and post-pollination (crossability) barriers by measuring the diploid progenitor’s anther lengths and by performing artificial cross experiments. One major finding was that the diploid progenitor’s ability for reproductive isolation might have markedly influenced the allopolyploid speciation of common wheat by causing lineage-associated patterns of gene flow. This and other findings revealed a possible important role of the genealogical and geographic structure of the parental species’ reproductive isolation in the origin of natural allopolyploid plants. The present study underscored the importance of approaches that incorporate information on the parental species’ reproductive isolation structure for studying the genetic and ecological basis of plant allopolyploid speciation.

## Study System

The diploid progenitor of common wheat, *Aegilops tauschii* Coss. (DD genome, formerly known as *Aegilops squarrosa* L.), is a wild wheat species that is primarily autogamous or geitonogamous. This species has a wide geographic range spanning from central Syria to western China in Eurasia^17,18^. In the currently accepted explanation of the origin of common wheat, male *Ae. tauschii* crossed with female *Triticum turgidum* L. (tetraploid, AABB genome) under natural conditions. This cross produced triploid F_1_ hybrids (ABD genome), which, in turn, produced hexaploid F_2_ hybrids (AABBDD genome), the direct ancestors of the extant allohexaploid common wheat. The hexaploid F_2_ arose through natural genome duplication via the union of unreduced gametes produced by the triploid F_1_ hybrids^19–21^. The allopolyploid speciation of common wheat was caused by human-mediated migration and/or habitat disturbance. Early in the evolution of common wheat, *Ae. tauschii* crossed with a cultivated form of *T. turgidum*, which arose from wild *T. turgidum* through domestication in the Fertile Crescent (ca. 10,000 years ago). The *T. turgidum* cultivar came into contact with *Ae. tauschii* through migration in association with the spread of agriculture across and beyond that region^22,23^. The exact place of the critical contact is not known. Molecular evidence suggested that allopolyploidisation was a recurring process during *T. aestivum* evolution^15,24^.

*Ae. tauschii* has distinctive genetic lineage structure that may facilitate intraspecific comparisons. This structure comprises two large lineages (TauL1 and TauL2), each including two sublineages (TauL1a and TauL1b within TauL1; TauL2a and TauL2b within TauL2), and one small lineage (TauL3)^25–28^. While TauL1 is geographically widespread within the species distribution range, TauL2 and TauL3 are restricted to the Transcaucasus-Middle East region and Georgia, respectively. Within the range of TauL1, TauL1a resides mainly in the western part and TauL1b in the eastern part. The ranges of TauL2a and TauL2b widely overlap, but TauL2a tends to occur in the western part of the range of TauL2, whereas the TauL2b mostly occurs in the eastern part. Genetically, TauL2 and TauL3 are much closer to the D genome of common wheat than TauL1 is, suggesting that the ancestors of TauL1 were probably not involved in the allopolyploid speciation of common wheat^15^. TauL2 seems to be the *Ae. tauschii* lineage that is closely related to, but not the closest sister of, common wheat’s D genome, because TauL2 and the common wheat D genome diverged long before the origin of common wheat, roughly 0.5 million years ago^15,29^. Interestingly, TauL1 and TauL2-TauL3 seem to be reproductively isolated in their natural habitats as, despite their wide overlap in the western part of the species range, the intermediate genotypes are rarely found^24^. A previous study identified two intraspecific groups of *Ae. tauschii* based on pollination-related traits: the short anther and long anther groups^30^. The short anther group consists of strong selfer accessions that produce a reduced amount of pollen (i.e., have low outcrossing potential). The long anther group consists of the accessions that are capable of facultative outcrossing and produce an increased amount of pollen (i.e., have high outcrossing potential).

## Results

### Anther length

**Figure 1:**
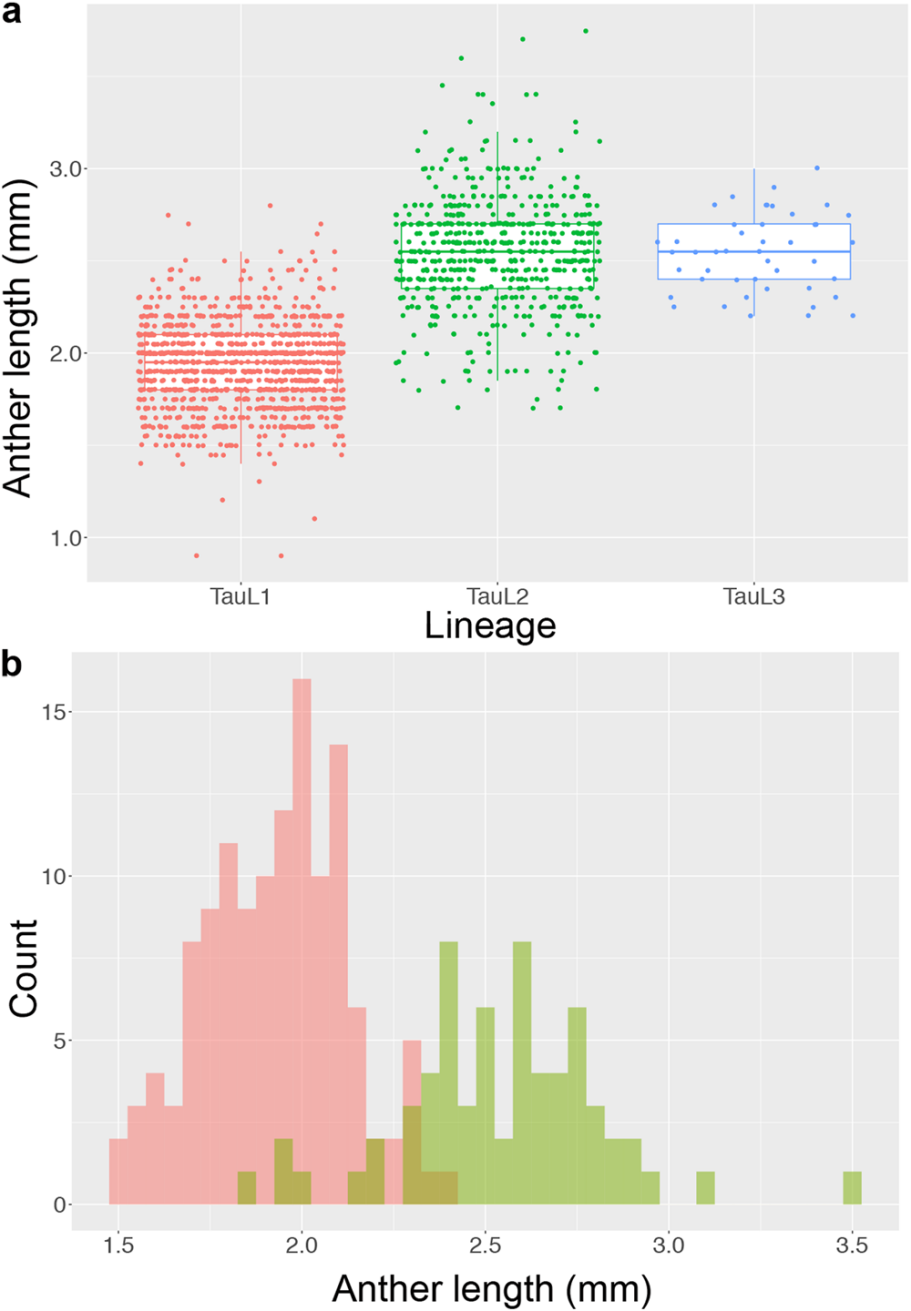
Anther length comparison among *Aegilops tauschii* lineages. (**a**) Lineage-wise box and dot plots of anther lengths. (**b**) Accession-wise histograms of mean anther length values of TauL1 (red) and TauL2 (light green). The TauL2 values that overlap with the TauL1 values are shown in dark green.

Because anther length is a good indicator of *Ae. tauschii*’s outcrossing potential, the anther length dataset obtained in our previous common garden experiment was used here to examine the outcrossing potential of the accessions^27,30^. Anthers were shorter in TauL1 than in TauL2 and TauL3 accessions. The mean length for TauL1 was 1.9 mm (1152 observations, standard deviation = 0.23, 128 accessions), whereas that for TauL2 and TauL3 was 2.6 mm (585 observations, standard deviation = 0.30, 65 accessions and 45 observations, standard deviation = 0.21, five accessions, respectively) (Fig. 1a). We examined if the lineage is a significant predictor of anther length by linear mixed model (LMM) analysis based on the TauL1 and TauL2 dataset (Supplementary Data S1). This analysis compared an alternative model that included the lineage as an explanatory variable to a null model that included no explanatory variable. Model selection was done based on the minimum Akaike Information Criterion (AIC) value, an estimate of information lost when a given model is used to approximate the process that generated observed data. The alternative LMM provided a positive coefficient (0.61) for TauL2, indicating that the anther lengths increased in this lineage. The inclusion of the explanatory variable (the lineage) as a fixed effect improved the goodness of prediction of the alternative model (AIC = −1630.3) relative to the null model (AIC = −1440.7) (Table 1). The departure of the alternative model from the null model was significant (likelihood ratio test, *P* < 2.2e-16), indicating that the lineage is a significant predictor of anther length in TauL1 and TauL2 accessions. The marginal *R* ^2^ (i.e., the proportion of variance explained by the fixed effect) for the alternative model was 0.56, whereas the conditional *R* ^2^ (i.e., the proportion of variance explained by the fixed and random effects) was 0.92. The accession-wise mean length histograms of TauL1 and TauL2 were largely separated, despite a minor overlap, and four TauL2 accessions (KU-2107, KU-2109, KU-2160, and KU-2124) had exceptionally short anthers (mean ≤ 2.0 mm) (Fig. 1b; Supplementary Data S1).

**Table 1:** Linear mixed models used to evaluate anther length difference between TauL1 and TauL2^a^.

		Model	
		Null	Alternative
Number of observations		1737	1737
Number of accessions		193	193
Coefficient of fixed effect	Intercept	2.14	1.93
	TauL2		0.61
*t* value for fixed effect	Intercept	81.68	98.75
	TauL2		18.10
Variance for random effect	Accession	0.13	0.04
	Spikelet within accession	8.5e–3	8.5e–3
Akaike Information Criterion		−1440.7	−1630.3
Likelihood ratio test (null model vs. alternative model)	*χ* ^2^		191.55
	Degree of freedom		1
	*P*		<2.2e–16
Marginal *R* ^2^			0.56
Conditional *R* ^2^			0.92

^a^Blank cells denote that the test was not applicable.

**Figure 2:**
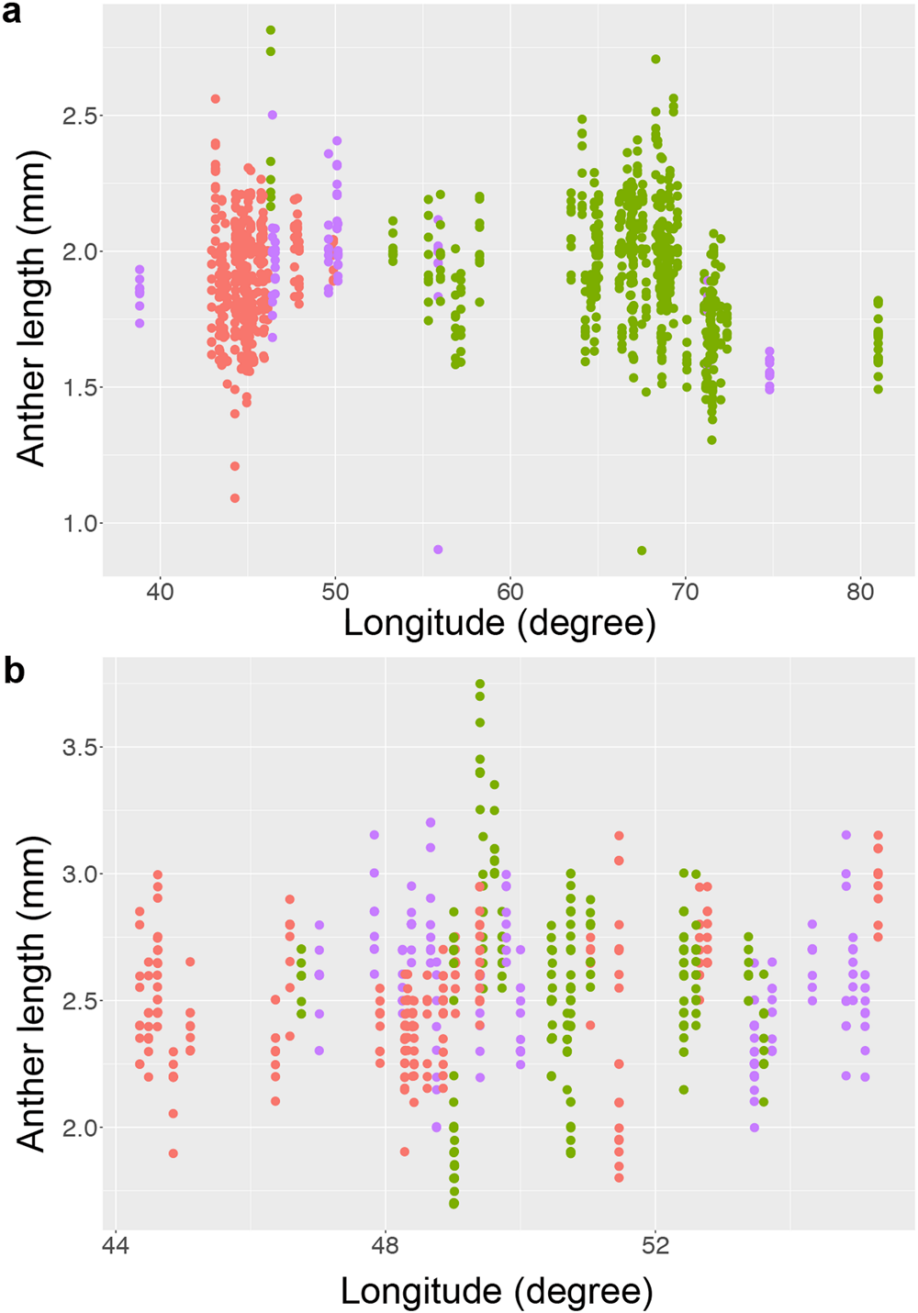
Anther length variation according to longitude. (**a**) Relationship between the longitude (*x*) and anther length (*y*) in TauL1. TauL1a and TauL2b are coloured in red and green, respectively. TauL1x (purple) is the intermediate form between TauL1a and TauL1b. (**b**) Relationship between the longitude (*x*) and anther length (*y*) in TauL2. TauL2a and TauL2b are coloured in red and green, respectively. TauL2x (purple) is the intermediate form between TauL2a and TauL2b.

Because each TauL1 and TauL2 has a west-east genetic structure, we further examined whether anther length was differentiated across the longitudinal distribution of each lineage. For this purpose, the intralineage relationships of anther length with the longitude of the localities of the accessions were analysed using the LMM-based model selection approach. Longitude was found to be a poor predictor of anther length in both TauL1 and TauL2 (Table 2; Fig. 2). In TauL1, longitude had a negative correlation with anther length (the fixed effect coefficient = −1.70e-03). The inclusion of longitude as a fixed effect in the alternative model did not improve the goodness of prediction in the alternative model (AIC = −1151.2) relative to the null model (AIC = −1151.7). The departure of the alternative model from the null model was not significant (likelihood ratio test, *P* = 0.23). The marginal *R* ^2^ for the alternative model was 8.27e-03, whereas the conditional *R* ^2^ was 0.77. In TauL2, longitude had a positive correlation with anther length (fixed effect coefficient = 0.01). The inclusion of longitude as a fixed effect slightly improved the goodness of prediction of the alternative model (AIC = −472.5) relative to the null model (AIC = −473.7). However, the departure of the alternative model from the null model was not significant (likelihood ratio test, *P* = 0.38). The marginal *R* ^2^ for the alternative model was 9.29e-03, whereas the conditional *R* ^2^ was 0.86.

**Table 2:** Linear mixed models used to evaluate the west-east differentiation of anther length in TauL1 and TauL2^a^.

		Model for TauL1		Model for TauL2	
		Null	Alternative	Null	Alternative
Number of observations		1098	1098	585	585
Number of accessions		122	122	65	65
Coefficient of fixed effect	Intercept	1.91	2.02	2.54	2.02
	Longitude		−1.70e-03		0.01
*t* value for fixed effect	Intercept	116.1	24.56	75.51	3.32
	Longitude		−1.21		0.87
Variance for random effect	Accession	0.03	0.03	0.07	0.07
	Spikelet within Accession	6.9e–3	6.9e-3	0.01	0.01
Akaike Information Criterion		−1151.7	−1151.2	−473.7	−472.5
Likelihood ratio test (null model vs. alternative model)	*χ* ^2^		1.46		0.76
	Degree of freedom		1		1
	*P*		0.23		0.38
Marginal *R* ^2^			8.27e-03		9.29e-03
Conditional *R* ^2^			0.77		0.86

^a^Blank cells denote that the test was not applicable.

### Crossability

**Figure 3:**
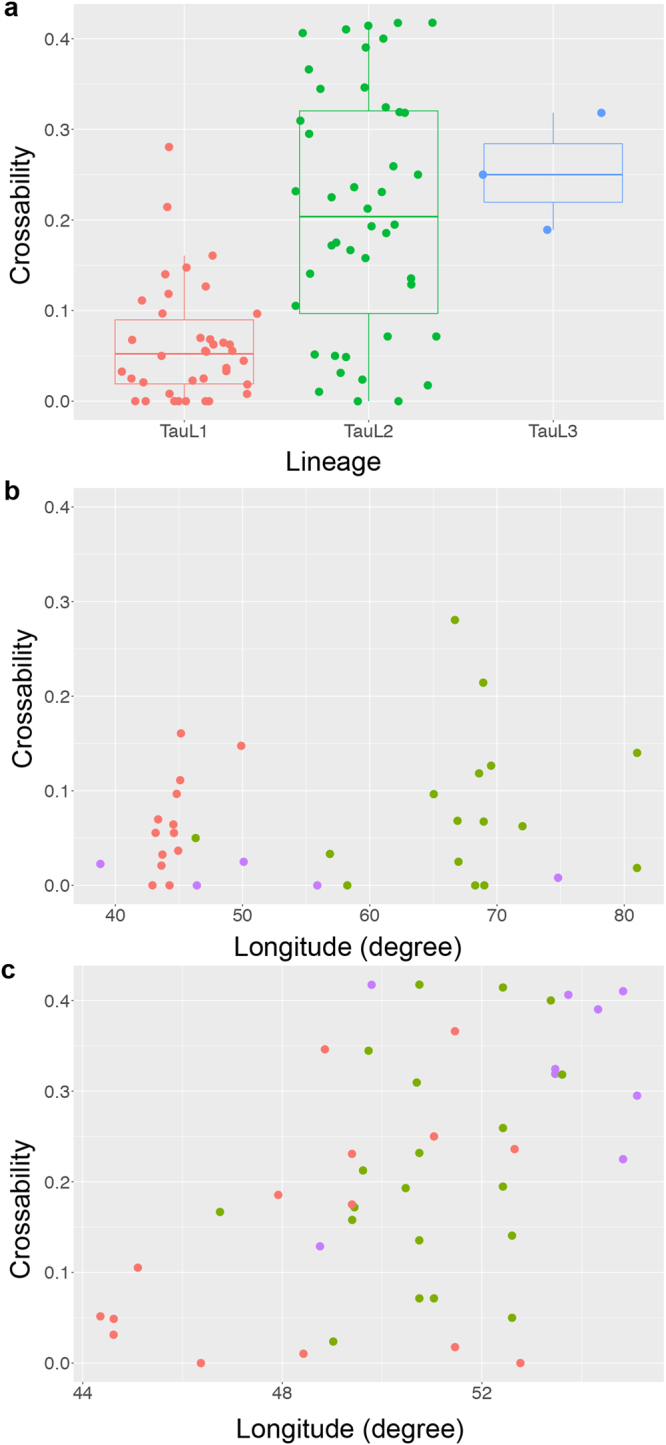
Genealogical and geographic structure of the crossability. (**a**) Lineage-wise box and dot plots of crossability. (**b**) Relationship between the longitude (*x*) and crossability (*y*) in TauL1. TauL1a and TauL2b are coloured in red and green, respectively. TauL1x (purple) is the intermediate form between TauL1a and TauL1b. (**c**) Relationship between the longitude (*x*) and crossability (*y*) in TauL2. TauL2a and TauL2b are coloured in red and green, respectively. TauL2x (purple) is the intermediate form between TauL2a and TauL2b.

Crossability was generally lower in TauL1 than in TauL2 and TauL3. It varied from 0.00 to 0.28 among the TauL1 accessions (mean = 0.06, standard deviation = 0.06; 38 accessions), from 0.00 to 0.42 among the TauL2 accessions (mean = 0.21, standard deviation = 0.13; 44 accessions), and from 0.19 to 0.32 among the TauL3 accessions (mean = 0.25, standard deviation = 0.06; three accessions) (Fig. 3a). We examined if the lineage is a significant predictor of crossability by generalised linear mixed model (GLMM) analysis based on the TauL1 and TauL2 dataset (Supplementary Data S2). This analysis compared an alternative model that included the lineage as an explanatory variable to a null model that included no explanatory variable. Model selection was done based on the minimum AIC value. The alternative GLMM provided a positive coefficient (1.61) for TauL2, indicating that the crossability increased in this lineage. The inclusion of lineage as a fixed effect improved the goodness of prediction of the alternative model (AIC = 1254.0) relative to the null model (AIC = 1281.3). The departure of the alternative model from the null model was significant (likelihood ratio test, *P* = 6.15e-08), indicating that lineage is a significant predictor of crossability in TauL1 and TauL2 accessions. The marginal *R* ^2^ for the alternative model was 0.11, whereas the conditional *R* ^2^ was 0.25 (Table 3).

**Table 3:** Generalised linear mixed models used to evaluate between-lineage differences regarding the crossability with the *Triticum turgidum* tester^a^.

		Model	
		Null	Alternative
Number of tester spikes crossed		236	236
Number of accessions		82	82
Coefficient of fixed effect	Intercept	−2.49	−3.37
	TauL2		1.61
*z* value	Intercept	−15.22	−15.46
	TauL2		5.85
Variance for random effect	Accession	1.45	0.80
	Spike	0.94	0.94
Akaike Information Criterion		1281.3	1254.0
Likelihood ratio test (null model vs. alternative model)	*χ* ^2^		29.32
	Degree of freedom		1
	*P*		6.15e–08
Marginal *R* ^2^			0.11
Conditional *R* ^2^			0.25

^a^Blank cells denote that the test was not applicable.

We further examined if crossability was differentiated across the longitudinal distribution of each TauL1 and TauL2 lineage using GLMM-based model selection approach. Longitude was a poor predictor of crossability in TauL1 (Table 4; Fig. 3). It was positively correlated with crossability (the fixed effect coefficient = 4.09e-3). However, its inclusion as a fixed effect did not improve the goodness of prediction of the alternative model (AIC = 306.3) relative to the null model (AIC = 304.4) (Table 4; Fig. 3b). The departure of the alternative model from the null model was not significant (likelihood ratio test, *P* = 0.81). The marginal and conditional *R* ^2^ for the alternative model was 4.96e-04. In contrast, longitude was a significant predictor of crossability in TauL2. Longitude had a positive correlation with crossability (the fixed effect coefficient = 0.24). The inclusion of longitude as a fixed effect in the alternative model improved the goodness of prediction of the alternative model (AIC = 885.8) relative to the null model (AIC = 899.3) (Table 4; Fig. 3c). The departure of the alternative model from the null model was significant (likelihood ratio test, *P* = 8.16e-05). The marginal *R* ^2^ for the alternative model was 0.09, whereas the conditional *R* ^2^ was 0.20.

**Table 4:** Generalised linear mixed models used to evaluate the west-east differentiation in the crossability with the *Triticum turgidum* tester in TauL1 and TauL2^a^.

		Model for TauL1		Model for TauL2	
		Null	Alternative	Null	Alternative
Number of tester spikes crossed		75	75	149	149
Number of accessions		34	34	44	44
Coefficient of fixed effect	Intercept	−3.47	−3.70	−1.75	−13.72
	Longitude		4.09e–3		0.24
*z* value for fixed effect	Intercept	–13.78	−3.76	−9.51	−4.93
	Longitude		0.24		4.33
Variance for random effect	Accession	0.00	0.00	1.05	0.58
	Spike	2.29	2.28	0.70	0.70
Akaike Information Criterion		304.4	306.3	899.3	885.8
Likelihood ratio test (null model vs. alternative model)	*χ* ^2^		0.06		15.52
	Degree of freedom		1		1
	*P*		0.81		8.16e–05
Marginal *R* ^2^			4.96e–04		0.09
Conditional *R* ^2^			4.96e–04		0.20

^a^Blank cells denote that the test was not applicable.

### Potential for natural hybridisation with *T. turgidum*

**Figure 4:**
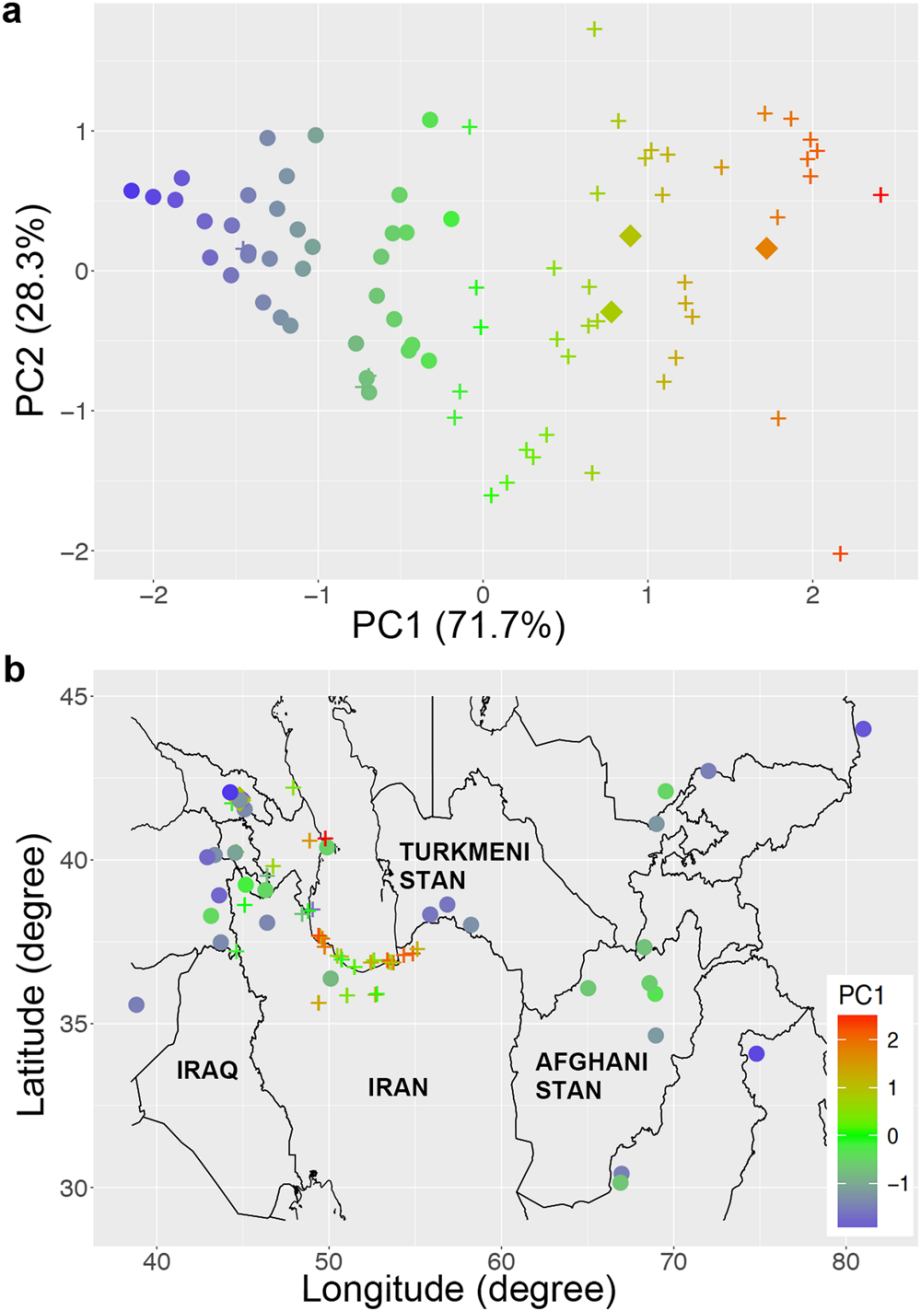
Genealogical and geographic structure of the variation of the potential for natural hybridisation with *Triticum turgidum* in the 82 *Aegilops tauschii* accessions. (**a**) Plot of the first (*x*) and second (*y*) principal components. Circles, crosses, and squares denote the TauL1, TauL2, and TauL3 accessions, respectively. Colour varies according to the scores of the first principal component (PC1). (**b**) Geographic distribution of the TauL1, TauL2, and TauL3 accessions. Circles, crosses, and squares denote TauL1, TauL2, and TauL3, respectively. Each accession is coloured according to its score along PC1. The six TauL1 accessions representing the adventive populations in the Shaanxi and Henan provinces are not shown. The map was drawn using the R software [R Core Team, R Foundation for Statistical Computing, Vienna, Austria, R: A Language and Environment for Statistical Computing., (2016) https://www.R-project.org (Date of access 01/06/2017)], the R package ‘ggplot2’ (R version 3.3.0) [*ggplot2: Elegant Graphics For Data Analysis*. (Springer-Verlag, 2009)] and a spatial dataset obtained from *DIVA-GIS* (version 7.5.0) [Hijmans, R. J., Guarino, L. & Mathur, P., *DIVA-GIS*., (2012) http://www.diva-gis.org/Data (Date of access: 01/03/2016)].

The mean anther length and crossability data were available for 82 accessions (Supplementary Data S3). The data for each of these accessions were used to infer its potential for natural hybridisation with *T. turgidum*. An index of those traits was produced by principal component analysis (PCA) based on the among-accessions correlation matrix. This index was the first principal component (PC1) in a two-dimensional space defined by the rectangular coordinate axes of mean anther length and crossability. The first and second principal components (PC1 and PC2) of the PCA explained 71.7% and 28.3% of the correlation structure between mean anther length and crossability, respectively (correlation coefficient = 0.43) (Fig. 4a). The PC1 was positively correlated with both mean anther length and crossability, as its eigenvectors and PC loadings were 0.71 and 0.85 for both characteristics, respectively. The eigenvectors of PC2 were −0.71 for the anther length mean (PC loadings = −0.53) and 0.71 for the crossability (PC loadings = 0.53). We assumed that long anther accessions (i.e., capable of facultative outcrossing) with high crossability are more likely to hybridise than short anther accessions (i.e., strong selfers) with low crossability. Based on this assumption, the PC1 provided a usable index for the potential of natural hybridisation with *T. turgidum*. While the PC1 scores of TauL1 were generally negative, those of TauL2 and TauL3 were mostly positive (Fig. 4a; Supplementary Data S3). Geographically, TauL2 accessions with particularly high PC1 scores (>1.5) clustered in the southern Caspian coast, except for the one located in the Azerbaijani coast (KU-2801) (Fig. 4b). One TauL3 accession (AE 454 from Georgia) had a PC1 score of 1.7.

## Discussion

TauL1 and TauL2 clearly differed in anther length (Fig. 1a,b; Table 1). No formal statistical analysis was performed for the TauL3 accessions, but they likely belonged to the long anther group (Fig. 1a). Thus, these results support Hammer’s grouping according to anther length, with TauL1 belonging to the short anther group and TauL2 and TauL3 belonging to the long anther group^30^. Moreover, these groups differ in other pollination-related traits, including the number of pollen grains per anther and the number of spikelets per spike^30^.

In grasses, the likelihood of outcrossing increases with increasing pollen-ovule ratios (i.e., the number of pollen grains per flower/the number of ovules per flower)^31^. *Aegilops tauschii* has one ovule per flower and anther length is positively correlated with the numbers of pollen grains per anther^30^. Therefore, outcrossing potential (i.e., the likelihood of outcrossing) is expected to be lower in TauL1 than in TauL2 and TauL3. In fact, TauL1 may represent an ecotype that prefers disturbed habitats where strong selfing (i.e., low likelihood of outcrossing) is adaptive^32^. This view is consistent with the fact that TauL1 accessions often show disturbed-habitat-related phenotypes such as high seed production ability, short vegetative growth span, and increased tolerance to abiotic stress^27,33^. In addition, *Ae. tauschii* expanded its distribution range from the Transcaucasus-Middle East region to Central Asia through migration of TauL1^27^. In a scenario where strong autogamy is a derivative state, the low anther length differentiation in TauL1 may suggest that the high-to-low shift in outcrossing potential might have occurred in the western part of the species range prior to the eastward migration (Table 2; Fig. 2a).

The strength of post-pollination isolation between TauL1 and TauL2 is very weak, and therefore, their F_1_ hybrids show no or modest reduction in fertility^34^. Thus, pre-pollination barriers may be the major mechanisms for the intraspecific reproductive isolation of TauL1 from TauL2. The low outcrossing potential of the TauL1 accessions might be a pre-pollination mechanism that reduces the amount of gene flow between TauL1 and TauL2 under natural conditions. Furthermore, the outcrossing potential might be involved in the interspecific reproductive isolation between *Ae. tauschii* and *T. turgidum* by limiting the likelihood of the contact between *Ae. tauschii* pollen and *T. turgidum* pistils. In this case, the pre-pollination reproductive isolation caused by the low outcrossing potential would be stronger between TauL1 and *T. turgidum* than between TauL2 and *T. turgidum*.

The cultivars of *T. turgidum* are genetically diverse. Nevertheless, only one tester was used in the cross experiment. For this reason, care must be taken when interpreting crossability patterns. Crossability with the tester was lower for TauL1 than for TauL2 accessions (Fig. 3a; Table 3). The tester was a durum wheat cultivar that belongs to a candidate subspecies (*T. turgidum* subsp. *durum*) for the female progenitor of common wheat^35^. Accordingly, the present study elucidates on the influence of *Ae. tauschii*’s ability to cause post-pollination reproductive isolation in the hybridisation with *T. turgidum* on the evolution of common wheat.

Molecular genetic evidence indicated that the allopolyploid speciation of common wheat involved the ancestors of TauL2 and TauL3^15^. However, the reason why the ancestors of TauL1 were not involved, although the geographic range of this lineage included the probable region of origin of common wheat, is not clear. One hypothesis is that the ancestral TauL1 was reproductively isolated from *T. turgidum*. This hypothesis is supported by differences between intraspecific lineages of *Ae. tauschii* regarding the expression of pre- and post-pollination barriers when crossed with *T. turgidum*: TauL1 expressed the examined barriers more strongly than TauL2 when crossed with the tester. Moreover, hybrid incompatibility phenotypes are more frequent in the tester-TauL1 F_1_ hybrids than in the tester-TauL2 F_1_ hybrids^36^, whereas the extent of unreduced gamete production does not markedly differ between the tester-TauL1 and tester-TauL2 F_1_ hybrids^15^.

The ancestors of TauL1 were probably strong selfers, like the current TauL1, and shed a reduced amount of pollen. Thus, the low outcrossing potential of the ancestral TauL1 might have acted as a pre-pollination barrier and limited the likelihood of their hybridisation with *T. turgidum*. Similarly, post-pollination reproductive isolation caused by crossability reduction and frequent expression of hybrid incompatibility might have been strong between the ancestors of TauL1 and *T. turgidum*. Accordingly, the ability of *Ae. tauschii* to cause reproductive isolation in the hybridisation with *T. turgidum* might have markedly influenced the allopolyploid speciation of common wheat by inducing lineage-associated patterns of gene flow.

Outcrossing potential reduction in TauL1 was 0.24 as compared with TauL2 [1-(TauL1 mean anther length/TauL2 mean anther length)]. Crossability reduction in TauL1 was 0.71 as compared with TauL2 [1-(TauL1 mean crossability/TauL2 mean crossability)]. The reduction in the post-pollination barrier was larger than in the pre-pollination barrier. However, the obtained values do not necessarily indicate that crossability contributed more than outcrossing potential to the exclusion of ancestral TauL1 from common wheat’s speciation. This is because the extent to which the reduction in outcrossing potential lowers the actual frequency of *Ae. tauschii* pollen grains’ arrival on the *T. turgidum* pistils is not known. In flowering plants, pre-zygotic barriers often contribute more to total reproductive isolation than post-zygotic barriers^37^. The outcrossing potential, possibly in association with other pre-pollination barriers such as niche differentiation, might have critically contributed to the exclusion of ancestral TauL1 from the allopolyploid speciation of common wheat.

In plants, reproductive isolation mechanisms are often polymorphic within species^38,39^. As this might be the case for *Ae. tauschii*, polymorphism in the reproductive isolation of the parental species may influence the patterns of allopolyploid speciation when this is genealogically and/or geographically structured. Thus, the present study highlighted that the structure of reproductive isolation within the parental species might have an important role in shaping the patterns of plant allopolyploid speciation. Clearly, a better understanding of the genetic and ecological basis of plant allopolyploid speciation requires approaches that incorporate the reproductive isolation structure of the parental species.

It is still not known if TauL2 and TauL3, both close relatives to the D genome of common wheat, have the reproductive phenotypes that may facilitate natural hybridisation with *T. turgidum*. The PCA based on mean anther length and crossability data showed that TauL2 and TauL3 have higher potential for the natural hybridisation than TauL1 (Fig. 4a). Furthermore, TauL2 accessions with particularly high hybridisation potential were found to cluster in the southern coastal region of the Caspian Sea (Fig. 4b). These findings provide phenotypic support for origin of D genome of common wheat from close relatives of TauL2. The clustering of the high hybridisation potential of TauL2 accessions is in striking accordance with the particularly short genetic distance to the common wheat D genome found for accessions exclusively located in the southwestern and southern Caspian coast of Iran^24^. The southern coastal region of the Caspian Sea is, therefore, a good candidate for the place of origin of common wheat. However, this remains unknown because the present study provided few clues about the genetic and phenotypic changes that *Ae. tauschii* might have undergone since the time of origin of common wheat. Further studies are required to assess whether the ancient *Ae. tauschii* was similar to the current *Ae. tauschii* in terms of lineage structure, geographic range, and potential for hybridisation with *T. turgidum*.

## Materials and Methods

### Plant materials

This study used 201 *Ae. tauschii* accessions representing the entire natural habitat range of the species and adventive Chinese populations (Supplementary Table S1). These accessions were randomly selected from a source collection, except for those originating from the peripheral habitats in Syria, India, and China. Each accession had a distinctive molecular marker genotype^27^. One hundred and thirty accessions belonged to TauL1, 66 to TauL2, and five to TauL3. One durum wheat cultivar, *Triticum turgidum* L. subsp. *durum* cv. ‘Langdon’, was used as tester in the artificial cross experiment. This subspecies is a candidate for the female progenitor of common wheat^35^.

### Measures of anther length and crossability

The anther length dataset contained the lengths of the three anthers located in the first florets of the central spikelets in the first, second, and third spikes (nine anthers per plant; one plant per accession) (Supplementary Data S1). These data were missing in two TauL1 (KU-2012 and KU-2809) and one TauL2 (KU-2078) accessions. The crossability of each accession with *T. turgidum* was evaluated through artificial crosses with the tester. This experiment was performed for 85 accessions: 38 TauL1, 44 TauL2, and three TauL3. *Ae. tauschii* and tester seeds were sown in early winter and plants grew in individual pots in a greenhouse. Crosses were then performed by applying the pollen of each *Ae. tauschii* accession to the pistils of the tester by hand. Spikes used in crosses were fully emasculated before anthesis and bagged individually. Two days after emasculation, the pistils of the first and second florets of the emasculated spikes (roughly, 20–40 florets per spike) were pollinated. Usually, pollen of one matured anther was applied to one or two pistils, and crosses used one to 15 tester spikes (median: two spikes) per *Ae. tauschii* accession (256 spikes in total). Spikes were immediately rebagged after pollination and ripened. No gibberellic acid solution was applied to the embryos after pollination. The same person performed emasculation and pollination to reduce the effect of possible technical idiosyncrasies. After harvest, the seeds set in each spike were counted and crossability was calculated for each *Ae. tauschii* accession as the number of well-developed seeds set divided by number of pollinated florets. Thus, both pre-zygotic (e.g., pollen-pistil incompatibility) and post-zygotic (e.g., embryo abortion) mechanisms might have influenced crossability values. The crossability data were compiled from published and unpublished observations (Supplementary Data S2)^40^.

### Statistical analysis

Differences between lineages regarding anther length and crossability were analysed by a model selection approach, based on LMM (anther length comparison) or GLMM (crossability comparison). These analyses were only performed for the TauL1 and TauL2 accessions, because TauL3 contained a small number of accessions. In each anther length and crossability analysis, we compared an alternative model that included the lineage as an explanatory variable to a null model that included no explanatory variable, using the lme4 package for R ver. 3.3^41,42^. The lmer function of the lme4 package was used in anther length comparisons to fit a fixed effect (lineage) and random effects (accession and spikelet nested within accession) with a Gaussian error distribution assumption based on a maximum likelihood estimation method. Comparisons were performed for 193 accessions. In the analysis of crossability differences, the glmer function of the lme4 package was used for fitting a fixed effect (lineage) and random effects (accession and spike) with a specific binomial error distribution on a logit link function. The significance of the departure of the alternative model from the null model was examined by a likelihood ratio test using the anova function. The marginal *R* ^2^ and conditional *R* ^2^ values of the alternative models were calculated using the r.squaredGLMM function of the MuMIn package for R ver. 3.3^43,44^.

The intralineage relationships of anther length and crossability with the longitude of the accessions’ localities were analysed using LMM-based (for anther length) or GLMM-based (for crossability) model selection approaches. The three accessions with missing anther length data were excluded from the LMM-based analysis. In addition, six TauL1 accessions (AT 47, AT 55, AT 60, AT 76, AT 80, and PI 508264) from far-east adventive localities were excluded from both LMM-based and GLMM-based analyses. Analytical procedures were identical to those described for the between-lineage comparisons, except that longitude, instead of lineage, was entered as a fixed effect into the alternative model structure. The bobyqa (for TauL1) and Nelder-Mead (for TauL2) optimizer options were invoked in the GLMM-based analysis. In all test procedures, differences were considered significant at *P* < 0.05.

A PCA based on the among-accessions correlation matrix was performed using the prcomp function of R ver. 3.3. A map showing the spatial distribution of the PC1 scores was created based on the geographic coordinates of the accessions, using a spatial dataset obtained from DIVA-GIS ver. 7.5.0 and the ggplot2 package for R ver. 3.3^45,46^.

### Data Availability

All data generated or analysed during this study are included in this published article (and its Supplementary Information files).

## Electronic supplementary material

Supplementary Information

Dataset S1

Dataset S2

Dataset S3
