# Supplementary material for: The role of reproductive isolation in allopolyploid speciation patterns: empirical insights from the progenitors of common wheat
Source: Sci Rep. 2017 Nov 22;7:16004. doi: 10.1038/s41598-017-15919-z (PMC5700127; doi:10.1038/s41598-017-15919-z)
Supplement: Supplementary file 1 — Supplementary Information [file 41598_2017_15919_MOESM1_ESM.pdf]

The role of reproductive isolation in allopolyploid speciation patterns: empirical insights from the progenitors of common wheat

Yoshihiro Matsuoka and Shigeo Takumi

Supplementary Information

Table S1 The *Ae. tauschii* accessions used. Source codes are IPK for Institut für Pflanzengenetik und Kulturpflanzenforschung, CGN for Centre for Genetic Resources, The Netherlands, ICARDA for International Center for Agricultural Research in the Dry Areas, KYOTO for Plant Germ-plasm Institute of Kyoto University, NBRP for National BioResources Project, OKAYAMA for Dr. Kenji Kato, Okayama University, and USDA for US Department of Agriculture.

No.	Accession	Country	Latitude	Longitude	Lineage	SubLineage	Source
1	AE 1037	Georgia	41.71	44.35	L2	L2a	IPK
2	AE 1038	Tajikistan	37.34	68.30	L1	L1b	IPK
3	AE 1090	Kazakhstan	42.10	69.53	L1	L1b	IPK
4	AE 454	Georgia	41.89	44.80	L3	L3	IPK
5	AE 457	Georgia	41.64	44.90	L3	L3	IPK
6	AE 929	Georgia	41.89	44.80	L3	L3	IPK
7	AE 933	Georgia	41.85	44.79	L1	L1a	IPK
8	AT 47	China	34.78	109.25	L1	L1a	OKAYAMA
9	AT 55	China	34.78	109.25	L1	L1b	OKAYAMA
10	AT 60	China	34.37	109.25	L1	L1b	OKAYAMA
11	AT 76	China	34.37	107.19	L1	L1b	OKAYAMA
12	AT 80	China	34.78	108.96	L1	L1b	OKAYAMA
13	CGN 10767	Pakistan	30.42	66.97	L1	L1b	CGN
14	CGN 10768	Pakistan	30.38	67.00	L1	L1b	CGN
15	CGN 10769	Pakistan	30.55	66.88	L1	L1b	CGN
16	CGN 10770	Pakistan	30.25	67.03	L1	L1b	CGN
17	CGN 10771	Pakistan	30.22	67.01	L1	L1b	CGN
18	IG 108561	Pakistan	29.88	66.83	L1	L1b	ICARDA
19	IG 120735	Turkmenistan	38.53	57.17	L1	L1b	ICARDA
20	IG 120736	Uzbekistan	39.92	66.37	L1	L1b	ICARDA
21	IG 120863	Dagestan	41.58	48.28	L2	L2a	ICARDA
22	IG 120866	Dagestan	41.88	48.38	L2	L2x	ICARDA
23	IG 123910	Uzbekistan	40.80	72.37	L1	L1b	ICARDA
24	IG 126273	Armenia	40.00	44.96	L1	L1a	ICARDA
25	IG 126280	Armenia	39.90	44.94	L1	L1a	ICARDA

26	IG 126293	Armenia	39.80	45.33	L1	L1a	ICARDA
27	IG 126353	Armenia	39.71	45.57	L1	L1a	ICARDA
28	IG 126387	Turkmenistan	38.64	56.86	L1	L1b	ICARDA
29	IG 126489	Turkmenistan	38.02	58.24	L1	L1b	ICARDA
30	IG 126991	Armenia	39.09	46.58	L2	L2a	ICARDA
31	IG 127015	Armenia	39.08	46.31	L1	L1b	ICARDA
32	IG 131606	Kyrgyzstan	42.72	72.01	L1	L1b	ICARDA
33	IG 46663	Pakistan	30.53	67.25	L1	L1b	ICARDA
34	IG 46666	Pakistan	30.75	67.55	L1	L1b	ICARDA
35	IG 46682	Pakistan	30.67	68.67	L1	L1b	ICARDA
36	IG 47173	Armenia	39.52	46.37	L2	L2a	ICARDA
37	IG 47182	Azerbaijan	39.05	48.67	L2	L2x	ICARDA
38	IG 47186	Azerbaijan	40.08	49.40	L2	L2x	ICARDA
39	IG 47188	Azerbaijan	40.98	47.83	L2	L2x	ICARDA
40	IG 47192	Azerbaijan	38.93	48.25	L2	L2x	ICARDA
41	IG 47193	Azerbaijan	38.75	48.40	L2	L2a	ICARDA
42	IG 47194	Azerbaijan	40.50	50.00	L2	L2x	ICARDA
43	IG 47196	Azerbaijan	40.38	49.88	L1	L1a	ICARDA
44	IG 47199	Azerbaijan	40.63	48.62	L2	L2a	ICARDA
45	IG 47202	Azerbaijan	39.80	46.75	L2	L2b	ICARDA
46	IG 47203	Azerbaijan	39.38	47.02	L2	L2x	ICARDA
47	IG 47204	Azerbaijan	41.20	49.03	L2	L2a	ICARDA
48	IG 47259	Syria	35.58	38.82	L1	L1x	ICARDA
49	IG 48042	India	34.08	74.80	L1	L1x	ICARDA
50	IG 48274	Dagestan	42.20	47.92	L2	L2a	ICARDA
51	IG 48508	Turkmenistan	38.33	55.87	L1	L1x	ICARDA
52	IG 48518	Turkmenistan	38.45	56.00	L1	L1b	ICARDA
53	IG 48539	Uzbekistan	41.10	69.00	L1	L1b	ICARDA
54	IG 48554	Tajikistan	39.47	67.50	L1	L1b	ICARDA
55	IG 48559	Tajikistan	39.75	68.63	L1	L1b	ICARDA
56	IG 48564	Tajikistan	40.08	69.12	L1	L1b	ICARDA
57	IG 48565	Uzbekistan	40.45	71.07	L1	L1b	ICARDA

58	IG 48567	Uzbekistan	40.57	71.70	L1	L1b	ICARDA
59	IG 48747	Armenia	40.28	44.63	L1	L1a	ICARDA
60	IG 48748	Armenia	40.18	44.67	L1	L1a	ICARDA
61	IG 48758	Armenia	40.25	44.33	L1	L1a	ICARDA
62	IG 49095	Iran	36.33	47.83	L1	L1a	ICARDA
63	KU-20-1	Dagestan	42.06	48.33	L2	L2a	KYOTO/NBRP
64	KU-20-10	Iran	37.04	50.69	L2	L2b	KYOTO/NBRP
65	KU-20-6	Pakistan	30.08	66.90	L1	L1b	KYOTO/NBRP
66	KU-20-7	Iran	35.85	51.04	L2	L2a	KYOTO/NBRP
67	KU-20-8	Iran	35.87	52.65	L2	L2a	KYOTO/NBRP
68	KU-20-9	Iran	36.88	53.47	L2	L2x	KYOTO/NBRP
69	KU-2001	Pakistan	30.15	66.90	L1	L1b	KYOTO/NBRP
70	KU-2003	Pakistan	30.15	66.90	L1	L1b	KYOTO/NBRP
71	KU-2006	Pakistan	30.69	66.67	L1	L1b	KYOTO/NBRP
72	KU-2008	Pakistan	31.03	66.33	L1	L1b	KYOTO/NBRP
73	KU-2010	Afghanistan	31.83	66.21	L1	L1b	KYOTO/NBRP
74	KU-2012	Afghanistan	32.03	66.69	L1	L1b	KYOTO/NBRP
75	KU-2016	Afghanistan	32.81	67.75	L1	L1b	KYOTO/NBRP
76	KU-2018	Afghanistan	33.80	68.41	L1	L1b	KYOTO/NBRP
77	KU-2022	Afghanistan	34.62	69.31	L1	L1b	KYOTO/NBRP
78	KU-2025	Afghanistan	35.91	68.92	L1	L1b	KYOTO/NBRP
79	KU-2027	Afghanistan	36.15	68.75	L1	L1b	KYOTO/NBRP
80	KU-2028	Afghanistan	36.18	68.65	L1	L1b	KYOTO/NBRP
81	KU-2032	Afghanistan	36.31	68.60	L1	L1b	KYOTO/NBRP
82	KU-2035	Afghanistan	36.22	68.59	L1	L1b	KYOTO/NBRP
83	KU-2039	Afghanistan	36.24	68.59	L1	L1b	KYOTO/NBRP
84	KU-2042	Afghanistan	36.20	68.52	L1	L1b	KYOTO/NBRP
85	KU-2044	Afghanistan	36.08	65.03	L1	L1b	KYOTO/NBRP
86	KU-2050	Afghanistan	36.01	64.79	L1	L1b	KYOTO/NBRP
87	KU-2051	Afghanistan	35.91	64.88	L1	L1b	KYOTO/NBRP
88	KU-2056	Afghanistan	35.95	64.90	L1	L1b	KYOTO/NBRP
89	KU-2058	Afghanistan	35.81	64.59	L1	L1b	KYOTO/NBRP

90	KU-2059	Afghanistan	35.74	64.27	L1	L1b	KYOTO/NBRP
91	KU-2063	Afghanistan	35.51	64.09	L1	L1b	KYOTO/NBRP
92	KU-2066	Afghanistan	35.25	63.46	L1	L1b	KYOTO/NBRP
93	KU-2068	Iran	36.38	50.09	L1	L1x	KYOTO/NBRP
94	KU-2069	Iran	35.85	51.04	L2	L2b	KYOTO/NBRP
95	KU-2074	Iran	36.88	53.47	L2	L2x	KYOTO/NBRP
96	KU-2075	Iran	36.87	53.73	L2	L2x	KYOTO/NBRP
97	KU-2076	Iran	37.10	54.33	L2	L2x	KYOTO/NBRP
98	KU-2077	Iran	37.20	54.93	L2	L2x	KYOTO/NBRP
99	KU-2078	Iran	37.14	54.83	L2	L2x	KYOTO/NBRP
100	KU-2079	Iran	37.14	54.83	L2	L2x	KYOTO/NBRP
101	KU-2080	Iran	37.27	55.11	L2	L2x	KYOTO/NBRP
102	KU-2082	Iran	37.17	55.31	L1	L1b	KYOTO/NBRP
103	KU-2083	Iran	37.10	55.30	L2	L2a	KYOTO/NBRP
104	KU-2086	Iran	35.92	52.77	L2	L2a	KYOTO/NBRP
105	KU-2087	Iran	36.74	53.29	L1	L1b	KYOTO/NBRP
106	KU-2088	Iran	36.92	53.38	L2	L2b	KYOTO/NBRP
107	KU-2090	Iran	36.86	53.61	L2	L2b	KYOTO/NBRP
108	KU-2091	Iran	36.92	52.60	L2	L2b	KYOTO/NBRP
109	KU-2092	Iran	36.92	52.60	L2	L2b	KYOTO/NBRP
110	KU-2093	Iran	36.87	52.42	L2	L2b	KYOTO/NBRP
111	KU-2096	Iran	36.87	52.42	L2	L2b	KYOTO/NBRP
112	KU-2097	Iran	36.87	52.42	L2	L2b	KYOTO/NBRP
113	KU-2098	Iran	36.95	50.75	L2	L2b	KYOTO/NBRP
114	KU-2100	Iran	37.07	50.47	L2	L2b	KYOTO/NBRP
115	KU-2101	Iran	37.17	50.46	L2	L2b	KYOTO/NBRP
116	KU-2103	Iran	37.34	49.73	L2	L2b	KYOTO/NBRP
117	KU-2104	Iran	37.59	49.62	L2	L2b	KYOTO/NBRP
118	KU-2105	Iran	37.66	49.44	L2	L2b	KYOTO/NBRP
119	KU-2106	Iran	37.67	49.40	L2	L2b	KYOTO/NBRP
120	KU-2107	Iran	38.19	49.01	L2	L2b	KYOTO/NBRP
121	KU-2108	Iran	38.19	49.01	L2	L2b	KYOTO/NBRP

122	KU-2109	Iran	38.49	49.02	L2	L2b	KYOTO/NBRP
123	KU-2110	Iran	38.43	48.76	L2	L2x	KYOTO/NBRP
124	KU-2111	Iran	38.35	48.42	L2	L2a	KYOTO/NBRP
125	KU-2112	Iran	38.26	48.29	L2	L2a	KYOTO/NBRP
126	KU-2113	Iran	36.76	45.94	L1	L1a	KYOTO/NBRP
127	KU-2115	Iran	37.07	45.74	L1	L1a	KYOTO/NBRP
128	KU-2116	Iran	38.29	45.02	L1	L1a	KYOTO/NBRP
129	KU-2118	Iran	38.62	45.10	L2	L2a	KYOTO/NBRP
130	KU-2120	Iran	38.49	45.88	L1	L1a	KYOTO/NBRP
131	KU-2121	Iran	38.38	46.13	L1	L1a	KYOTO/NBRP
132	KU-2122	Iran	38.08	46.41	L1	L1x	KYOTO/NBRP
133	KU-2124	Iran	36.72	51.46	L2	L2a	KYOTO/NBRP
134	KU-2126	Iran	36.72	51.46	L2	L2a	KYOTO/NBRP
135	KU-2131	Turkey	38.29	43.15	L1	L1a	KYOTO/NBRP
136	KU-2132	Turkey	38.29	43.15	L1	L1a	KYOTO/NBRP
137	KU-2133	Turkey	38.64	43.83	L1	L1a	KYOTO/NBRP
138	KU-2136	Turkey	38.92	43.62	L1	L1a	KYOTO/NBRP
139	KU-2137	Turkey	39.07	43.54	L1	L1a	KYOTO/NBRP
140	KU-2138	Turkey	39.04	43.51	L1	L1a	KYOTO/NBRP
141	KU-2140	Turkey	39.04	43.51	L1	L1a	KYOTO/NBRP
142	KU-2141	Turkey	38.94	43.41	L1	L1a	KYOTO/NBRP
143	KU-2142	Iran	39.32	44.84	L1	L1a	KYOTO/NBRP
144	KU-2143	Iran	39.26	45.07	L1	L1a	KYOTO/NBRP
145	KU-2144	Iran	39.24	45.16	L1	L1a	KYOTO/NBRP
146	KU-2145	Iran	39.10	45.24	L1	L1a	KYOTO/NBRP
147	KU-2148	Iran	38.75	45.90	L1	L1a	KYOTO/NBRP
148	KU-2149	Iran	38.90	45.76	L1	L1a	KYOTO/NBRP
149	KU-2150	Iran	38.80	45.77	L1	L1a	KYOTO/NBRP
150	KU-2151	Iran	37.44	47.67	L1	L1a	KYOTO/NBRP
151	KU-2152	Iran	37.15	47.93	L1	L1a	KYOTO/NBRP
152	KU-2153	Iran	36.33	50.15	L1	L1x	KYOTO/NBRP
153	KU-2154	Iran	35.99	49.61	L1	L1x	KYOTO/NBRP

154	KU-2155	Iran	35.63	49.40	L2	L2a	KYOTO/NBRP
155	KU-2156	Iran	35.63	49.40	L2	L2a	KYOTO/NBRP
156	KU-2157	Iran	34.16	46.56	L1	L1x	KYOTO/NBRP
157	KU-2158	Iran	36.95	50.75	L2	L2b	KYOTO/NBRP
158	KU-2159	Iran	36.95	50.75	L2	L2b	KYOTO/NBRP
159	KU-2160	Iran	36.95	50.75	L2	L2b	KYOTO/NBRP
160	KU-2612	Afghanistan	34.64	68.96	L1	L1b	KYOTO/NBRP
161	KU-2617	Afghanistan	36.64	70.07	L1	L1b	KYOTO/NBRP
162	KU-2619	Afghanistan	37.19	71.27	L1	L1x	KYOTO/NBRP
163	KU-2621	Afghanistan	37.19	71.24	L1	L1b	KYOTO/NBRP
164	KU-2624	Afghanistan	36.88	71.39	L1	L1b	KYOTO/NBRP
165	KU-2627	Afghanistan	36.80	71.16	L1	L1b	KYOTO/NBRP
166	KU-2630	Afghanistan	37.02	71.60	L1	L1b	KYOTO/NBRP
167	KU-2632	Afghanistan	36.98	71.60	L1	L1b	KYOTO/NBRP
168	KU-2633	Afghanistan	36.93	71.45	L1	L1b	KYOTO/NBRP
169	KU-2635	Afghanistan	36.88	71.55	L1	L1b	KYOTO/NBRP
170	KU-2636	Afghanistan	36.62	71.50	L1	L1b	KYOTO/NBRP
171	KU-2638	Afghanistan	36.57	71.54	L1	L1b	KYOTO/NBRP
172	KU-2639	Afghanistan	36.40	69.11	L1	L1b	KYOTO/NBRP
173	KU-2801	Azerbaijan	40.66	49.79	L2	L2x	KYOTO/NBRP
174	KU-2804	Azerbaijan	40.59	48.85	L2	L2a	KYOTO/NBRP
175	KU-2806	Azerbaijan	40.59	48.85	L2	L2a	KYOTO/NBRP
176	KU-2809	Armenia	40.25	44.62	L1	L1a	KYOTO/NBRP
177	KU-2810	Armenia	40.25	44.62	L1	L1a	KYOTO/NBRP
178	KU-2811	Armenia	40.25	44.62	L2	L2a	KYOTO/NBRP
179	KU-2814	Armenia	40.25	44.62	L1	L1a	KYOTO/NBRP
180	KU-2816	Armenia	40.22	44.55	L1	L1a	KYOTO/NBRP
181	KU-2821	Armenia	40.20	44.75	L1	L1a	KYOTO/NBRP
182	KU-2822A	Armenia	40.39	44.27	L1	L1a	KYOTO/NBRP
183	KU-2823	Armenia	40.38	44.30	L1	L1a	KYOTO/NBRP
184	KU-2824	Armenia	40.38	44.30	L1	L1a	KYOTO/NBRP
185	KU-2826	Georgia	41.55	45.10	L1	L1a	KYOTO/NBRP

186	KU-2827	Georgia	41.76	44.85	L2	L2a	KYOTO/NBRP
187	KU-2828	Georgia	41.84	44.94	L1	L1a	KYOTO/NBRP
188	KU-2829A	Georgia	41.82	44.82	L3	L3	KYOTO/NBRP
189	KU-2832	Georgia	41.82	44.82	L3	L3	KYOTO/NBRP
190	KU-2834	Georgia	41.82	44.82	L1	L1a	KYOTO/NBRP
191	KU-2835B	Georgia	42.09	44.48	L2	L2a	KYOTO/NBRP
192	KU-2836	Georgia	42.07	44.26	L1	L1a	KYOTO/NBRP
193	PI 476874	Afghanistan	36.08	65.03	L1	L1b	USDA
194	PI 486267	Turkey	37.20	44.62	L2	L2a	USDA
195	PI 486270	Turkey	37.78	44.33	L1	L1a	USDA
196	PI 486274	Turkey	40.15	43.37	L1	L1a	USDA
197	PI 486277	Turkey	40.08	42.93	L1	L1a	USDA
198	PI 499262	China	44.00	81.00	L1	L1b	USDA
199	PI 508262	China	44.00	81.00	L1	L1b	USDA
200	PI 508264	China	34.00	114.00	L1	L1b	USDA
201	PI 554319	Turkey	37.48	43.72	L1	L1a	USDA
